# The effect of long working hours on 10-year risk of coronary heart disease and stroke in the Korean population: the Korea National Health and Nutrition Examination Survey (KNHANES), 2007 to 2013

**DOI:** 10.1186/s40557-016-0149-5

**Published:** 2016-11-15

**Authors:** Dong-Wook Lee, Yun-Chul Hong, Kyoung-Bok Min, Tae-Shik Kim, Min-Seok Kim, Mo-Yeol Kang

**Affiliations:** 1 Department of Preventive Medicine, College of Medicine, Seoul National University, 103 Daehangno, Jongno-gu, Seoul, 110-799 Republic of Korea; 2 Institute of Environmental Medicine, Seoul National University Medical Research Center, 103 Daehangno, Jongno-gu, Seoul, 110-799 Republic of Korea

**Keywords:** Coronary heart disease, Stroke, Long working hours, Korean national health and nutrition examination survey

## Abstract

**Background:**

Recently, the emergence of long working hours and the associated conditions such as coronary heart disease (CHD) and stroke have gained attention. The aim of this study was to investigate the association between long working hours and the 10-year-risk of CHD and stroke, estimated by Jee’s health risk-appraisal model for ischemic heart disease.

**Methods:**

We analyzed data from Koreans who randomly enrolled in Korean National Health and Nutrition Examination Survey 2008–2012 and finally included 13,799 participants. The participants were classified as per their working hours: 0–30 h/week, 31–39 h/week, 40 h/week, 41–50 h/week, 51–60 h/week, 61–70 h/week, 71–80 h/week, and >80 h/week. The risks for CHD and stroke were determined using Jee’s health risk-appraisal model. Multiple logistic regression was used to analyze the association between working hours and 10-year risk for CHD.

**Results:**

The 10-year risks for CHD and stroke were significantly and positively associated with working hours in both men and women. Furthermore, higher risks for CHD and stroke were associated with longer working hours in women.

**Conclusion:**

Long working hours are significantly associated with the risks of CHD and stroke, estimated by Jee’s health risk-appraisal model. This study suggests the need for proper management of working hours to reduce CHD risk and stroke risk in the Korean population.

**Electronic supplementary material:**

The online version of this article (doi:10.1186/s40557-016-0149-5) contains supplementary material, which is available to authorized users.

## Background

Long working hours can cause health problems such as hypertension, musculoskeletal discomfort, diabetes, occupational injury, increased suicide rate, sleep problems, preterm birth, poor psychological health, and unhealthy lifestyle conditions [1, 2]. Recently, the emergence of long working hours and the associated conditions such as coronary heart disease (CHD) and stroke, have gained attention [3–6]. However, the association of long working hours with CHD and stroke has not yet been clearly established. A recently published meta-analysis study reported that long working hours could be associated with CHD, but evidence for a dose-response relationship between working hours and CHD was insufficient, despite sufficient evidence for its association with stroke [7].

In the Korean population, studies have shown that long working hours may increase the risk of CHD, especially in people who work extremely long hours [8]. However, a limitation of this study was the use of the Framingham equation to determine the 10-year risk; this equation is derived from the participants mainly comprising middle-income Caucasians.

However, the risk could be overestimated in Asian population when estimating the risk by the Framingham equation model [9]. Ahn et al. suggested that the Framingham equation model overestimates the risk of ischemic heart disease in the Korean population [10]. Therefore, estimation of the accurate risks of CHD and stroke is warranted to investigate the relationship of long working hours with CHD and stroke in Korea.

To overcome the limitation of the Framingham equation model in Korean populations, Jee et al. developed an individualized health risk-appraisal model for ischemic heart disease and stroke, by using well-known risk factors such as hypertension and hypercholesterolemia, using data derived from the Korean population. Outcomes were ascertained from data on the National Health Insurance Corporation claim and death certificate. They used Cox proportional hazard model when developing health-appraisal model to estimate the 10-year risk for ischemic heart disease and stroke. They applied split-half method that the first half of data was used for developing a model and the rest was used for testing for validity of models. This health-appraisal model accurately predicted the actual rates of events [11, 12]. The actual ischemic heart disease event rates were similar to the event rates predicted by the Korean risk prediction model for ischemic heart disease [13].

The primary aim of this study was to investigate the association between long working hours and risks of CHD and stroke estimated by Jee’s health risk-appraisal model. In this assessment, differences between genders were taken in account. Considering that the risks of CHD and stroke are preventable, it is important to understand the characteristics of these events, specific risk factors, and populations at risk. Therefore, this study also aimed to identify specific risk factors for CHD and stroke, which may be associated with working hours.

## Methods

### Study design and participants

The current study used data derived from the Korean National Health and Nutrition Examination Survey (KNHANES) 2007–2013. The KNHANES is a cross-sectional, nationally representative survey, conducted by the Korean Centers for Disease Control and Prevention (KCDC). Multistage probability sampling, stratified according to geographic location, gender, and age, was used. It collects information about the participants’ socioeconomic status, anthropometric measures, health interview, health examination, and nutrition survey. All participants of the KNHANES used in the current study provided written informed consent. This nationwide survey was approved by the Institutional Review Board (IRB) of the KCDC (IRB: 2007-02-CON-04-P; 2008-04EXP-01-C; 2009-01CON-03-2C; 2010-02CON-21-C; 2011-02CON-06-C; 2012-01EXP-01-2C; 2013-07CON-03-4C).

**Fig. 1:**
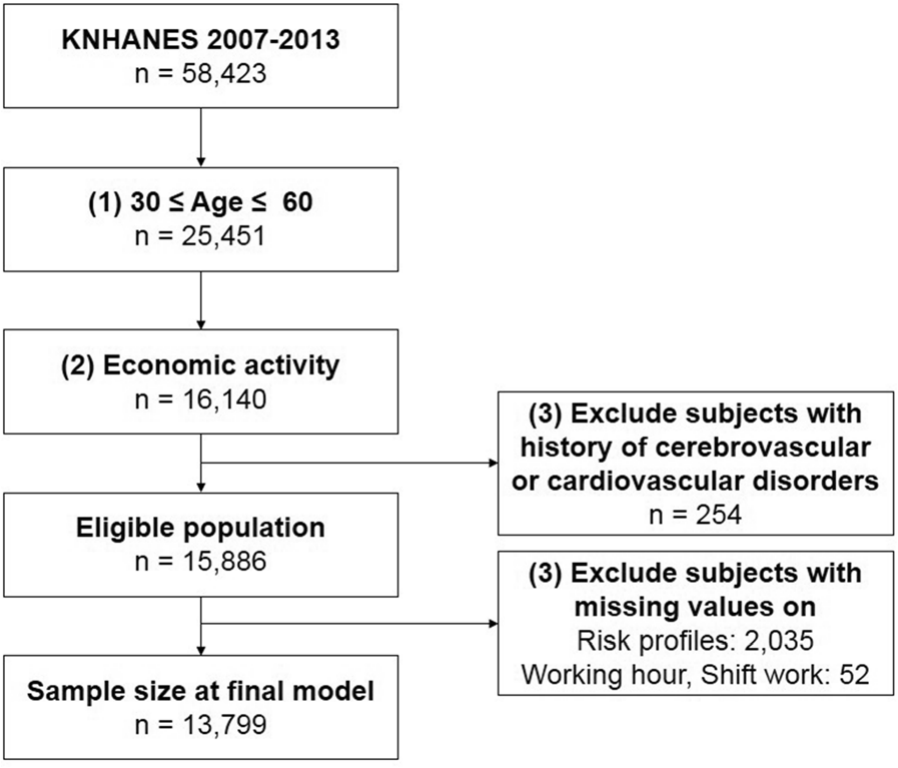
Schematic diagram of the number of participants

In total, 58,423 individuals (24,871 individuals from the 4^th^ KNHANES, 25,534 individuals from the 5^th^ KNHANES, and 8,018 individuals from the 6^th^ KNHANES) participated in the surveys. For our analysis, we excluded participants who were younger than 30 years of age and older than 60 years of age. Because Jee’s health-appraisal models for CHD and stroke were developed using Korean individuals between the age of 30 years - 60 years of age, we only included those age groups in our analysis (*n* = 32,972). Subjects who were unemployed or lacked information on economic activity (*n* = 9,311) as well as subjects with a history of cerebrovascular or cardiovascular disorders (*n* = 254) were excluded. The risk for CHD was calculated by combining 8 risk factors, and the risk for stroke was calculated by combining 8 risk factors. Subjects with missing values were also excluded (2,035 subjects were eliminated due to absence of one value for these risk factors and 52 subjects were eliminated due to missing information on shift work or working hours). Finally, of 15,886 eligible participants, the final sample size for analysis was 13,799 (7,565 men and 6,234 women) after excluding subjects with missing values (Fig. 1).

### Data collection

The KNHANES survey included questions about a wide array of characteristics. We used the following variables: age, gender, occupation-related variables, chronic disease diagnosis, and health behaviour. Completed questionnaires were reviewed by trained staff and entered into a database.

Working hours were measured as the actual number of hours the respondent worked per week without mealtime at all jobs. According to the Korean labour law, standard working hours are 40 h/week, and the reference group included people who worked 40 h/week. Working-hour groups were defined as 0–30 h/week, 31–39 h/week, 40 h/week, 41–50 h/week, 51–60 h/week, 61–70 h/week, 71–80 h/week, and >80 h/week. Occupations were classified into 6 groups: “managers and professionals,” “office workers,” “service and sales workers,” “agriculture, forestry and fisheries workers,” “craft, device, and machine operators, and assembly workers,” and “manual workers.” Daytime work is a full-time schedule in which subjects usually worked between 6 am and 6 pm, excluding rotators. The shift-work group included any schedule other than daytime work such as day-night shift, fixed night, regular rotation, 24-h shift, split shift, and irregular shifts. Smoking status was categorized as non-smoker, ex-smoker, and current smoker. In order to estimate the risk with Jee’s health-appraisal model, the alcohol consumption level at the interview was calculated as consumed alcohol in grams per day. Physical inactivity was defined as engaging in ≥10 min of mild physical activity less than 5 times per week.

Height and weight were measured during the physical examination. Body mass index (BMI) was calculated as weight in kilograms divided by height in meters squared. BMI was classified into 2 groups: <25 kg/m^2^ and ≥25 kg/m^2^. Blood pressure (BP) was measured in the right arm at the level of the heart using a standard mercury sphygmomanometer (Baumanometer; WA Baum, Copiague, NY, USA) while the subjects were seated and after they had rested for 5 min. The average of 2 systolic and diastolic blood pressure (SBP and DBP, respectively) readings, which were recorded at an interval of 5 min, was used for analysis. After a 12-h overnight fast, blood samples were obtained from the subjects through an antecubital vein. Total cholesterol (TC) and high-density lipoprotein (HDL) were measured using an ADVIA 1650 autoanalizer (Bayer, Tarrytown, NY, USA) from 2007 to 2008, and Hitachi Automatic Analyzer 7600 (Hitachi, Japan) from 2009 to 2012. Therefore, we used the corrected value of HDL by using conversion formula suggested by KCDC. The participants were considered to have diabetes mellitus (DM) if they met one of the following conditions: (1) fasting plasma glucose level ≥ 126 mg/dL, (2) medical diagnosis of DM by a trained medical professional, or (3) treatment with oral hypoglycemic agents or insulin injections.

### Estimation of the 10-year risks for CHD and stroke

Individuals’ risk for CHD was determined using Jee’s health risk-appraisal model for ischemic heart disease, which is based on data collected nationwide from health screenings, cause of death statistics, and insurance claims. This is the first CHD risk-prediction model in Korea that incorporated risk predictors of age, TC, HDL, SBP, DBP, smoking status, and diabetes by gender. After development, the model was validated in another random sample representative of the Korean population. Individuals’ 10-year risk for stroke was also estimated using Jee’s health risk-appraisal model for stroke, which is based on similar methods of the model for CHD. This model estimated the 10-year risk of stroke in Koreans by using age, SBP, diabetes, smoking status, physical inactivity, BMI, alcohol consumption, and total cholesterol by gender. To estimate the future risk of CHD and stroke, we calculated the number of points for each risk factor, after which the individual’s 10-year risk was computed using risk functions for Korean men and women, separately. As both model for CHD and model for stroke was validated by comparing actual event and prediction of event according to decile of predicted risk in each sex, we defined cases as high-risk group when the each risk was ≥90 percentile in each sex. To evaluate the association between weekly working hours and high risk for CHD, and weekly working hours and high risk for stroke, the odds ratios (ORs) and 95% confidence intervals (CIs) were calculated after adjustment for household income, employment condition, occupation, and work shift.

### Statistical analysis

The basic characteristics were described by means and standard deviation (SD) or frequencies with percentages for men and women, separately. Risk profiles used for estimating risks for CHD and stroke were presented as means and SD, and categorical values were presented as frequencies and percentage by each gender. First, we performed nonparametric analyses of the associations between weekly working hours and 10-year risks for CHD and stroke to visualize the association. After adjustment for household income, employment condition, occupation, and shift work, generalized additive models considering Poisson distribution were constructed for each outcome using the gam package of R version 2.12.2 (R Foundation for Statistical Computing, Vienna, Austria). Subsequently, the average 10-year risks for CHD and stroke by working-hour schedule were calculated for each gender. Finally, multiple logistic regression was conducted to assess the association between working hours and 10-year risks for CHD and stroke, estimated by Jee’s health risk-appraisal model. As these risks differ according to weekly working hours, we compared the 40 h/week group (reference group) with other working-hour groups. As the risk function used to estimate 10-year risks for CHD and stroke already considered age, SBP, and any other risk profiles, these variables were not used for adjusting variables. After adjustment for household income, employment condition, occupation, work shift, and weekly working hours, multiple logistic regression was performed using PROC LOGISTIC of SAS version 9.3 (SAS Institute, Cary, NC, USA). Two-tailed *p*-values < 0.05 were considered statistically significant. Additionally, the nonparametric analyses to visualize the association and multiple logistic regression were performed by stratifying 2 age group: <45y and ≥45y.

## Results

Table 1 shows the occupational characteristics of the study population. Among the 13,799 participants, 7,565 (54.8%) were men and 6,234 (45.2%) were women. The proportion of paid workers was 61.7% among men and 61.5% among women. In addition, among women, 13.7% were unpaid family workers. In both male and female subjects, <25% of the subjects did shift work, and 25.1% of male and 17.3% of female subjects worked >60 h/week.

**Table 1:** Characteristics of the study population

	Men		Women	
	*n*	%	*n*	%
Age (years)				
30–39	2475	32.7	1785	28.6
40–49	2661	35.2	2249	36.1
50–60	2429	32.1	2200	35.3
Education				
Below elementary school	546	7.2	1041	16.7
Middle school graduate	771	10.2	872	14.0
High school graduate	2802	37.0	2487	39.9
Above college graduate	3446	45.6	1834	29.4
Income status				
1st Quartile	437	5.8	545	8.7
2nd Quartile	1733	22.9	1573	25.2
3rd Quartile	2518	33.3	1993	32.0
4th Quartile	2877	38.0	2123	34.1
Employment condition				
Paid worker	4671	61.7	3836	61.5
Self-employed or employer	2743	36.3	1547	24.8
Unpaid family worker	151	2.0	851	13.7
Occupation				
Managers and professionals	1822	24.1	1298	20.8
Office workers	1264	16.7	819	13.1
Service and sales workers	1193	15.8	2025	32.5
Agriculture, forestry, and fishery workers	576	7.6	553	8.9
Craft, device, and machine operators and assembly workers	2188	28.9	411	6.6
Manual workers	522	6.9	1128	18.1
Work shift				
Fixed-day schedule	6392	84.5	5144	82.5
Any schedule other than fixed-day schedule^*^	1173	15.5	1090	17.5
Weekly working hours				
< 30	460	6.1	1357	21.8
30–40	649	8.6	974	15.6
40	1406	18.6	981	15.7
40–50	1567	20.7	1085	17.4
50–60	1587	21.0	757	12.1
60–70	1009	13.3	488	7.8
70–80	555	7.3	367	5.9
≥ 80	332	4.4	225	3.6
Total	7565	54.8	6234	45.2

^*^Any schedule other than fixed-day schedule includes day-night shift, fixed night, regular rotation, 24-h shift, split shift, irregular shift, and another schedule

A summary of the each component for Jee’s health risk-appraisal model according to weekly working hours for each gender is presented in Table 2. The mean age (SD) of the male and female subjects was 44.56 (±8.43 SD) and 45.22 (±8.26 SD) years. The levels of TC were similar in men and women, while the BP was slightly lower in women. Likewise, the other risk factors for CHD, such as prevalence of diabetes, current smoking, and alcohol consumption were higher among male subjects than among female subjects. Generally, the level of each variable increased as the weekly work hours increased from 40 h/week to >80 h/week, while those who worked 30–40 h/week and <30 h/week showed higher values than those who worked 40 h/week.

**Table 2:** Cardiovascular and stroke risk profiles

Weekly working hours	SBP		DBP		TC		HDL		Physical Inactivity		DM		Smoking		BMI		Drinking	
	Mean	SD	Mean	SD	Mean	SD	Mean	SD	*n*	%	*n*	%	*n*	%	Mean	SD	*n*	%
Men																		
< 30	122.33	16.1	81.90	11.4	193.37	35.4	48.62	12.7	247	53.7	54	11.7	230	50.0	24.50	3.1	358	77.8
30–40	120.90	14.6	81.88	10.3	194.21	35.3	50.03	12.0	317	48.8	64	9.9	318	49.0	24.44	2.9	520	80.1
40	118.97	14.3	81.20	10.8	191.79	33.0	48.15	10.9	720	51.2	120	8.5	616	43.8	24.51	2.9	1164	82.8
40–50	119.04	14.5	80.72	10.6	193.68	36.5	49.04	11.4	783	50.0	115	7.3	738	47.1	24.34	3.1	1260	80.4
50–60	119.06	14.2	80.77	10.4	191.08	33.6	48.75	11.3	797	50.2	115	7.2	775	48.8	24.35	3.1	1301	82.0
60–70	118.95	14.9	80.65	10.8	192.61	34.4	49.28	11.2	507	50.2	77	7.6	508	50.3	24.24	3.1	813	80.6
70–80	119.80	15.0	80.90	11.0	192.00	35.4	48.79	12.7	252	45.4	49	8.8	283	51.0	24.69	3.0	446	80.4
≥ 0.	120.77	14.9	81.50	10.6	197.04	36.3	48.17	10.2	157	47.3	44	13.3	171	51.5	24.37	3.1	269	81.0
Total	119.51	14.7	81.03	10.7	192.69	34.7	48.85	11.5	3780	50.0	638	8.4	3639	48.1	24.41	3.0	6131	81.0
Women																		
< 30	112.91	15.8	74.12	10.0	190.26	35.3	55.69	12.4	717	52.8	62	4.6	71	5.2	23.39	3.4	544	40.1
30–40	113.71	16.0	74.48	10.2	190.95	35.4	55.79	12.4	492	50.5	41	4.2	55	5.6	23.29	3.5	433	44.5
40	111.85	15.0	74.00	9.7	184.69	33.9	56.17	12.2	487	49.6	44	4.5	45	4.6	23.05	3.2	436	44.4
40–50	113.69	16.4	74.83	10.8	187.96	35.4	56.25	12.5	557	51.3	43	4.0	58	5.3	23.27	3.3	501	46.2
50–60	115.42	16.7	75.18	10.1	190.20	34.0	55.34	12.4	393	51.9	39	5.2	53	7.0	23.62	3.3	360	47.6
60–70	115.38	16.3	74.89	10.4	191.65	35.2	55.69	13.2	245	50.2	26	5.3	35	7.2	23.74	3.3	216	44.3
70–80	118.29	17.9	76.83	11.1	193.66	36.9	54.60	11.6	156	42.5	23	6.3	28	7.6	24.02	3.4	172	46.9
6.8	117.88	17.0	76.57	10.1	199.32	39.7	55.43	12.6	98	43.6	16	7.1	14	6.2	24.50	3.2	106	47.1
Total	114.00	16.2	74.72	10.3	189.72	35.3	55.76	12.4	3145	50.4	294	4.7	359	5.8	23.43	3.4	2768	44.4

*SBP* systolic blood pressure, *DBP* diastolic blood pressure, *TC* total cholesterol, *HDL* high density lipoprotein, *DM* diabetes mellitus, *BMI* body mass index

**Fig. 2:**
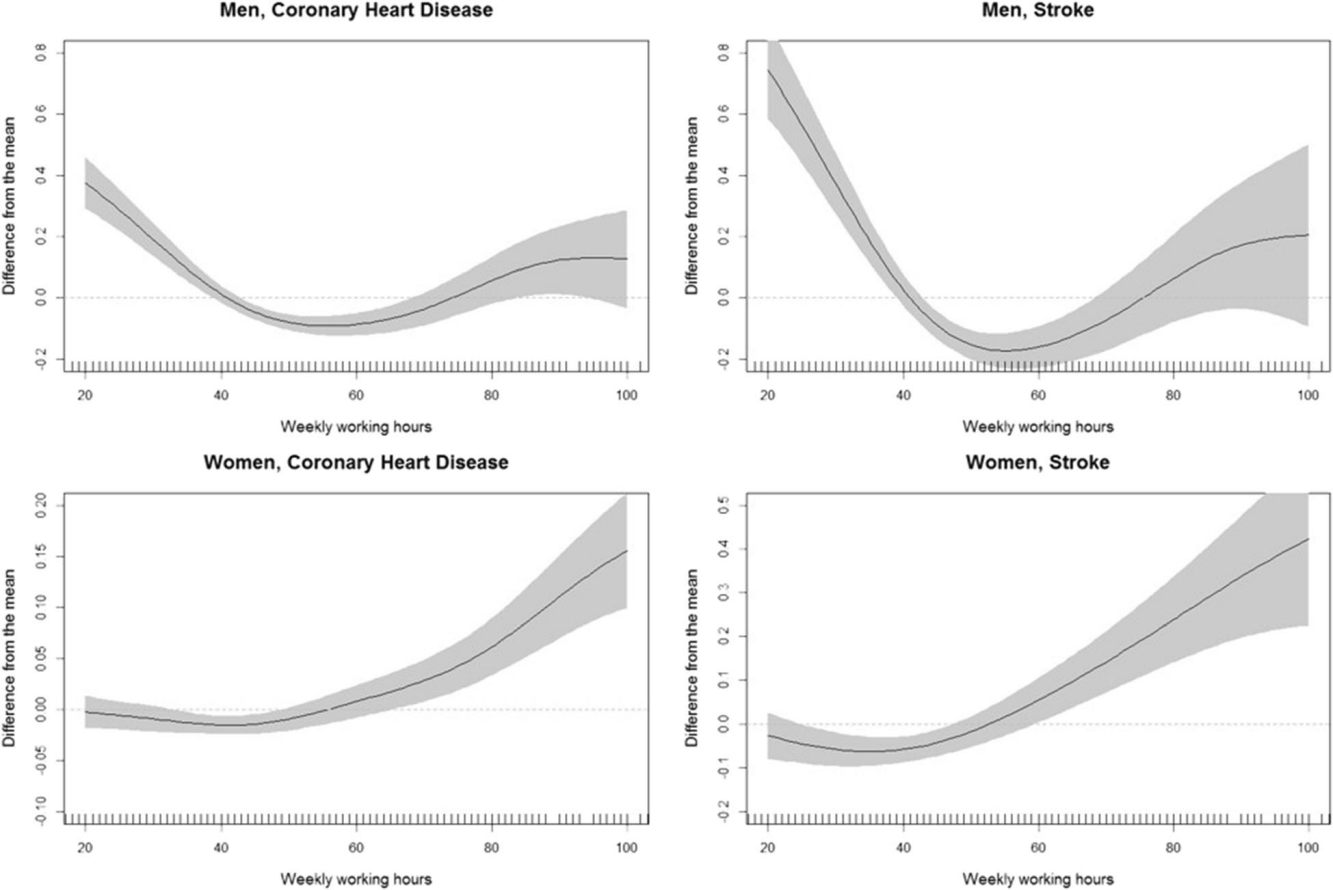
Nonlinear association between weekly working hours, risk for CHD, and risk for stoke in men and women

The nonparametric associations between weekly working hours and 10-year risks for CHD and stroke were evaluated for each gender (Fig. 2). Ten-year risks of both CHD and stroke increased with increasing weekly working hours in subjects working >50 h/week, while decreased with increasing weekly working hours in subjects working <40 h/week.

The risk of CHD and stroke, estimated by Jee’s health risk-appraisal model, is presented according to weekly working hours in Table 3. The average risk of CHD predicted by Jee’s model was 1.11% and 0.26% in male and female subjects, respectively, and that of stroke was 1.97% and 1.45% in male and female subjects, respectively. On comparing those who worked 40 h/week and those who worked >80 h/week, the ORs for high risk of CHD were 1.76 (95% CI: 1.23–2.52) in men and 1.63 (95% CI: 1.03–2.58) in women. The ORs for high risk of stroke were 1.49 (95% CI: 1.03–2.16) in women who worked between 50 and 60 h/week and 2.32 (95% CI: 1.46–3.69) in women who worked >80 h/week, as compared to women who worked 40 h/week. Trend tests were performed after excluding participants who worked <40 h/week. A statistically significant dose-response relationship was found between weekly working hours and high risk for stroke among female subjects.

**Table 3:** Odds ratios for high 10-year risk of CHD and stroke

Weekly working hours			Estimated 10-year CHD risk		≥90th percentile of estimated risk of CHD					Estimated 10-year stroke risk		≥90th percentile of estimated risk of stroke				
	*n*	%	mean	SD	*n*	%	aOR^a^	95% CI		mean	SD	*n*	%	aOR^a^	95% CI	
Men																
< 30	460	6.1	1.59	14.7	89	19.4	**1.75**	**1.28**	**2.40**	1.59	14.7	97	21.1	**1.77**	**1.29**	**2.42**
30–40	649	8.6	1.25	19.3	72	11.1	1.04	0.76	1.42	1.25	19.3	86	13.3	1.24	0.91	1.68
40	1406	18.6	1.05	5.4	122	8.7	1.00	Reference		1.05	5.4	115	8.18	8.2	Reference	
40–50	1567	20.7	1.03	13.2	137	8.7	0.89	0.69	1.16	1.03	13.2	130	8.3	0.85	0.65	1.11
50–60	1587	21.0	1.01	36.0	137	8.6	0.87	0.67	1.12	1.01	36	127	8.0	0.79	0.60	1.03
60–70	1009	13.3	1.06	46.0	88	8.7	0.83	0.62	1.11	1.06	46	93	9.2	0.87	0.65	1.17
70–80	555	7.3	1.13	14.4	52	9.4	0.83	0.59	1.19	1.13	14.4	63	11.4	0.99	0.70	1.39
.39	332	4.4	1.37	4.9	59	17.8	**1.76**	**1.23**	**2.52**	1.37	4.86	45	13.6	1.23	0.83	1.82
Total	7565	100.0	1.11	21.8	756	10.0				1.97	15	756	10.0			
*p* trend							0.43							0.94		
Women																
< 30	1357	21.8	0.26	18.2	136	10.0	1.11	0.81	1.54	1.45	10.2	141	10.4	**1.41**	**1.00**	**1.99**
30–40	974	15.6	0.26	122.0	95	9.8	1.08	0.77	1.53	1.42	6.2	96	9.9	1.34	0.94	1.93
40	981	15.7	0.19	16.8	65	6.6	1.00	Reference		1.14	26.5	53	5.40	5.4	Reference	
40–50	1085	17.4	0.23	106.0	88	8.1	1.01	0.71	1.43	1.33	28.8	84	7.7	1.18	0.82	1.71
50–60	757	12.1	0.28	24.6	81	10.7	1.06	0.74	1.51	1.59	10	91	12.0	**1.49**	**1.03**	**2.16**
60–70	488	7.8	0.29	14.5	61	12.5	1.09	0.74	1.61	1.65	10.1	60	12.3	1.31	0.87	1.98
70–80	367	5.9	0.33	8.5	58	15.8	1.32	0.88	1.97	1.81	2.4	54	14.7	1.47	0.96	2.25
.25	225	3.6	0.4	37.3	39	17.3	**1.63**	**1.03**	**2.58**	2.06	7.5	44	19.6	**2.32**	**1.46**	**3.69**
Total	6234	100.0	0.26	0.3	623	10.0				1.45	1.4	623	10.0			
*p* trend							0.06							0.01		

*CHD* coronary heart disease

^a^adjusted for household income, employment condition, occupation, work shift, and weekly working hour; values displaying significant differences are given in bold (*p*-value ≤ 0.05)

*p for trend was tested after excluding participants with <40 weekly working hours

In stratified analysis by age, the nonlinear association was weaker in younger age group (<45y), while similar in older age group (≥45y) (Additional file 1: Figure S1, S2). Long working hours (80 h/week) were associated with the high risk of CHD in men (OR: 1.85, 95% CI: 1.25–2.75) and associated with the high risk of stroke in women (OR: 1.65, 95% CI: 1.01–2.67) (Additional file 2: Table S1).

## Discussion

The results of our study show that the 10-year risks for CHD and stroke were significantly associated with working hours. Furthermore, higher risks for CHD and stroke were associated with longer working hours in women. Among the male participants, compared to those who worked 40 h/week, the longest working-hour group showed statistically significant differences in high risk for CHD. However, no such difference was found high risk for stroke. In addition, among female participants, the longest working-hour group showed higher OR for both CHD and stroke.

After stratified by age group, the nonparametric analyses in older age group (≥45y) were consistent with the analyses in main result, while the nonparametric analyses in younger age group (<45y) show weaker association in younger age group. Among older age group, it was statistically significant that the association between the longest working-hours and the risk of CHD in men and between the longest working-hours and the risk of stroke in women.

In our study, compared to the regular working hours, the ORs of high risk for CHD in the longest working-hour group (≥80 h/week) were 1.76 in men and 1.63 in women and were statistically significant. Although our results shows significant associations only in longest working-hour group, these results are in line with those of previous studies, which reported an association between long working hours and CHD. A meta-analysis study concluded that longer working hours was related with the risk of CHD with an OR of 1.37 [14]. Another meta-analysis of 4 prospective studies reported a relative risk of 1.39 for long working hours on CHD [4]. However, recent meta-analysis of large data performed by Kivimaki et al. reported that there was no statistically significant association between working 55 h per week or longer and coronary heart disease [7]. Further studies to investigate how long working hours is associated with coronary heart disease are necessary.

Although several studies have reported an association between long working hours and CHD risk, only a few studies have investigated the association between long working hours and stroke. For example, in a census-based longitudinal study in the United Kingdom, O’reilly and Rosato reported that those who worked ≥55 h/week showed a higher risk of stroke, but the difference was not statistically significant [5]. In the Korean population, however, a case-control study of 940 cases of incident hemorrhagic stroke concluded that longer regular working time and extended duration of strenuous activity during work could increase the risk of hemorrhagic stroke [15]. Recent multi-national studies using unpublished studies reported that long working hours was associated with stroke in a dose-response relationship. We also found a statistically significant association between long working hours and stroke. The ORs for stroke in the longest working-hour group were remarkable (1.22 in men and 1.48 in women), but statistically significant values were found only in women.

We defined high risks for CHD and stroke when the risk for each disease was higher than or equal to the 90th percentile of risk in each gender while considering differences in the risk-estimating methods and risk profiles, and weekly working hours as per gender. Among women, longer working hours showed a statistically significant association with the risk for stroke and marginally significant association with the risk for CHD (*p* for trend = 0.01 and 0.06, respectively). However, we did not find any such statistically significant association among men. It is possible that these contrasting findings reflect gender differences in influences on the cardiovascular system according to social role. Incomplete recovery from work is known to be associated with death by cardiovascular disease and the employee’s imbalance between work and family responsibilities. Complete recovery away from the workplace is an important component for preventing CHD and stroke [16, 17]. In eastern countries, married women are expected to do unpaid family work such as childcare, cleaning, preparing meals, and caring for elders, regardless of the individual’s working condition. In these cultures, female employees who work longer hours could be overloaded with unpaid family work. These excessive demands could cause excessive stress or lack of time spent on health promotion.

The results of the generalized additive model to nonlinear association suggest a difference in the direction of the association between participants who worked <40 h/week and those who worked ≥40 h/week. Furthermore, we observed a positive association between reduced working hours (<30 h/week) and risk of CHD and stroke in men, and between reduced working hours and risk of stroke in women. One possible explanation of the result is that the workers working less than 40 h per week, which is legal working hours in the Korea, may have inferior socioeconomic status and working condition comparing to the workers working 40 h or longer. Those who have short working hours per week are probably precarious workers and non-regular workers in the Korea, and they could have relatively large work stress from their position [18]. Another explanation may be that selection process between those who works longer hours and those who works reduced hours. In our study, participants with short working hours had higher SBP, DBP, total cholesterol level, physical inactivity, diabetes prevalence. It means that the possibility that those who reduced working hours had poorer health status than those who works general hours (40 h/week) or long hours. As people who have disease would not work long hours, health problem could affect how long time people works. Unfortunately, our cross-sectional study design cannot identify in which direction the selection process between working hours and risks for CHD and stroke may have occurred. Therefore, further studies with a longitudinal design are needed.

Several studies have suggested that long working hours are positively correlated with risk factors for CHD and stroke. The association between work hours and self-reported hypertension has been reported in California [19]. In Japan, increased BP and heart rate were reported among those who periodically worked overtime [20]. In a meta-analysis, a positive association between longer working hours and type 2 diabetes was reported [21]. An individual’s lifestyle such as smoking or frequent drinking was also associated with longer working hours [22, 23]. In our study, we observed higher SBP and DBP among subjects with longer working hours in both men and women (Table 2). Higher TC, higher prevalence of diabetes, and higher prevalence of smoking were seen in those with longer working hours. Compared to those who worked 40 h/week and those who worked ≥80 h/week, the prevalence of diabetes (8.53% vs. 13.25%, respectively) and the percentage of smokers (43.81% vs. 51.51%, respectively) showed significant differences among men, whereas the TC (184.69 mg/dl vs. 199.32 mg/dl, respectively), body mass index (23.05 kg/m2 vs. 24.50 kg/m2, respectively), and prevalence of diabetes (4.49% vs. 7.11%) showed significant differences among women.

Regarding its association with CHD and stroke, working hours could affect an individual’s health through various mechanisms. First, the person’s physical recovery may remain incomplete. Long working hours are associated with reduced time for recovery and limited relaxation time after work. Chronic health impairment could be caused by incomplete physical recovery due to prolonged exposure to work demands such as long working hours [24]. An effect-recovery model suggested that work effort draws upon one’s resources, thereby causing strain reaction [25]. Considering that long working hours could be associated with high job demands, employees who spend more time in the workplace could be restrained and may not be able to avail of appropriate relaxation time to reduce their strain [26, 27]. Second, work-life imbalance could be caused by long working hours. Bad family relationships are have been shown to be associated with sickness-related absence, psychological distress, and poor health [28]. These associations may be more important to Korean women who tend to be the primary care-giver of the family. As such, long working hours could have a larger effect on women’s health because of the domestic burden. In our study, women showed a dose-response relationship between weekly working hours and risks for CHD and stroke (Table 3). Similarly, in Japan, long working hours was associated with poor social dysfunction and poor general health among female factory employees [29]. Third, long working hours could adversely affect an individual’s health, causing physical inactivity, longer time spent in the sitting position, or risky drinking, which are risk factors for CHD or stroke [30–32].

Our study has some strengths. As the results were derived from a representative sample of the general Korean population, we could investigate the population with better variance by using a large sample size with longer working hours than that used in other studies. In addition, by using Jee’s health-appraisal model that was developed and validated in the Korean population for predicting 10-year risk for CHD and stroke, we could more accurately predict the risk as compared to previous studies. Moreover, accurate measurements of BP, TC, and HDL support the validity of the estimated 10-year risks for CHD and stroke.

Despite these strengths, there are limitations to this study. The results from the cross-sectional data preclude us from commenting on causal inferences. To overcome this issue, a longitudinal study design using established outcomes as recorded CHD or stroke event should be performed. Furthermore, we could not consider other factors that may affect the association between working hours, and CHD and stroke, such as job-demand level, poor mental health, and social support. Considering these limitations, further studies are required to confirm our findings.

## Conclusion

Long working hours increase the risks of CHD and stroke. These associations were more significantly observed among women than men, especially for the risk of stroke. These results suggest the need for proper management of working hours to reduce the incidence of CHD and stroke in the Korean population. We hope that this study will contribute to the improvement in labor management and reduce the risks of CHD and stroke that are associated with long working hours.

## Additional files

Additional file 1: Figure S1. Nonlinear association between weekly working hours, risk for CHD, and risk for stroke, stratified by age (<45y). **Figure S2.** Nonlinear association between weekly working hours, risk for CHD, and risk for stroke, stratified by age (≥45y). (DOCX 463 kb)
Additional file 2: Table S1. Odds ratios for high 10-year risk of CHD and stroke among participants ≥ 45y. (DOCX 35 kb)

## Abbreviations

BMI: Body mass index
BP: Blood pressure
CHD: Coronary heart disease
DBP: Diastolic blood pressure
DM: Diabetes mellitus
HDL: High-density lipoprotein
KCDC: Korean centers for disease control and prevention
KNHANES: Korean national health and nutrition examination survey
SBP: Systolic blood pressure
TC: Total cholesterol
