# Supplementary material for: The effect of long working hours on 10-year risk of coronary heart disease and stroke in the Korean population: the Korea National Health and Nutrition Examination Survey (KNHANES), 2007 to 2013
Source: Ann Occup Environ Med. 2016 Nov 15;28:64. doi: 10.1186/s40557-016-0149-5 (PMC5111276; doi:10.1186/s40557-016-0149-5)
Supplement: Additional file 1: Figure S1. — Nonlinear association between weekly working hours, risk for CHD, and risk for stroke, stratified by age (<45y). Figure S2. Nonlinear association between weekly working hours, risk for CHD, and risk for stroke, stratified by age (≥45y). (DOCX 463 kb) [file 40557_2016_149_MOESM1_ESM.docx]

**Figure S1**. Nonlinear association between weekly working hours, risk for CHD, and risk for stroke, stratified by age (<45y)


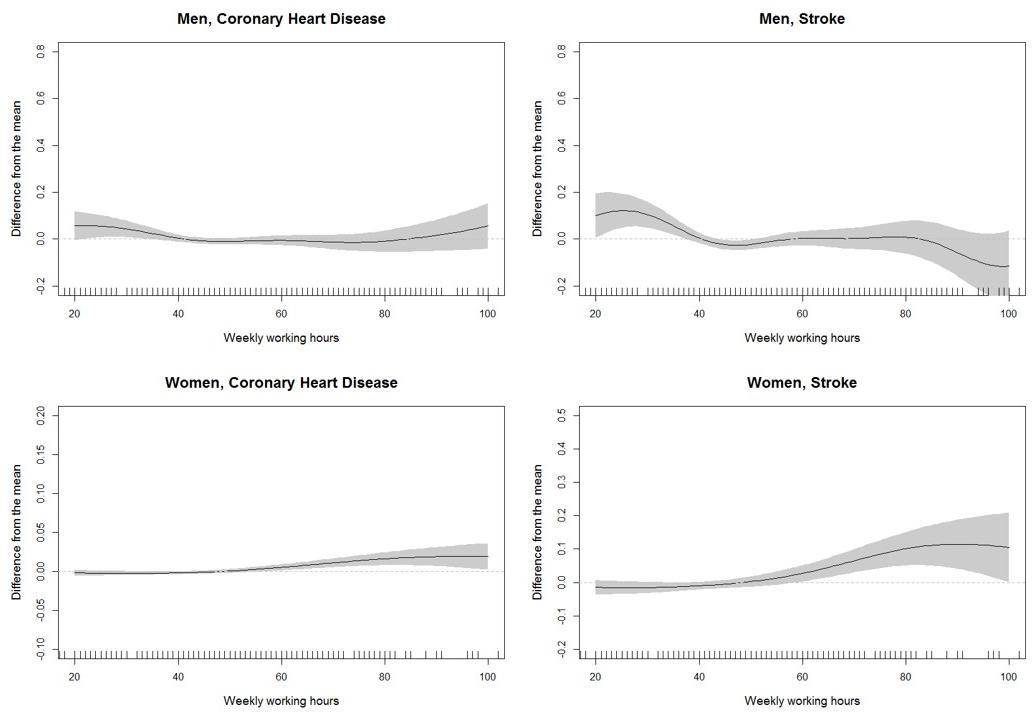


Nonparametric associations of weekly working hours with calculated 10-year risks for CHD and stroke. Data were adjusted for household income, employment condition, occupation, and shift work.

**Figure S2.** Nonlinear association between weekly working hours, risk for CHD, and risk for stroke, stratified by age (≥45y)


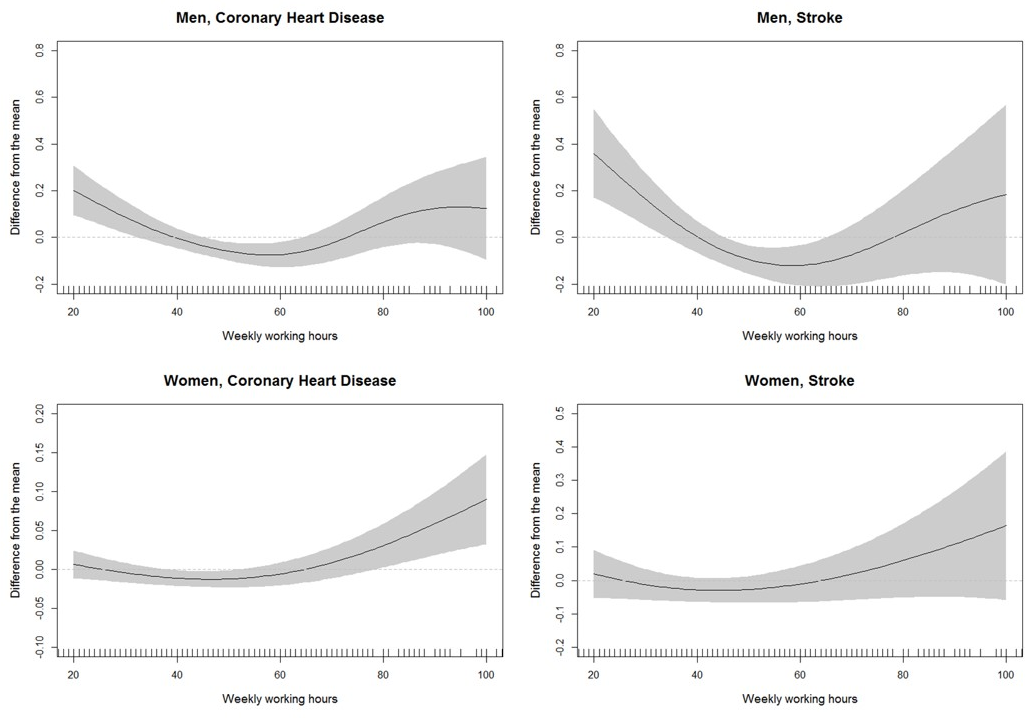


Nonparametric associations of weekly working hours with calculated 10-year risks for CHD and stroke. Data were adjusted for household income, employment condition, occupation, and shift work.
