# Supplementary material for: The effect of long working hours on 10-year risk of coronary heart disease and stroke in the Korean population: the Korea National Health and Nutrition Examination Survey (KNHANES), 2007 to 2013
Source: Ann Occup Environ Med. 2016 Nov 15;28:64. doi: 10.1186/s40557-016-0149-5 (PMC5111276; doi:10.1186/s40557-016-0149-5)
Supplement: Additional file 2: Table S1. — Odds ratios for high 10-year risk of CHD and stroke among participants ≥ 45y. (DOCX 35 kb) [file 40557_2016_149_MOESM2_ESM.docx]

**Additional file 2: Table S1**. Odds ratios for high 10-year risk of CHD and stroke among participants≥45y

| Weekly working hours |  |  |  | Estimated 10-year CHD risk | |  | ≥90th percentile of estimated risk of CHD | | | | |  | Estimated 10-year stroke risk | |  | ≥90th percentile of estimated risk of stroke | | | | |
| --- | --- | --- | --- | --- | --- | --- | --- | --- | --- | --- | --- | --- | --- | --- | --- | --- | --- | --- | --- | --- |
|  | n | % |  | mean | SD |  | n | % | aOR^§^ | 95% CI | |  | mean | SD |  | n | % | aOR^§^ | 95% CI | |
| **Male** |  |  |  |  |  |  |  |  |  |  |  |  |  |  |  |  |  |  |  |  |
| <30 | 333 | 9.1 |  | 1.96 | 1.4 |  | 88 | 26.4 | **1.52** | **1.09** | **2.12** |  | 3.78 | 2.7 |  | 97 | 29.1 | **1.49** | **1.07** | **2.07** |
| 30–40 | 372 | 10.1 |  | 1.76 | 1.2 |  | 70 | 18.8 | 1.01 | 0.72 | 1.42 |  | 3.31 | 2.1 |  | 85 | 22.9 | 1.20 | 0.86 | 1.66 |
| 40 | 639 | 17.4 |  | 1.63 | 1.0 |  | 115 | 18.0 | 1 | Referent | |  | 3.01 | 2.3 |  | 115 | 114 | 1 | Referent | |
| 40–50 | 696 | 19.0 |  | 1.66 | 1.1 |  | 130 | 18.7 | 1.02 | 0.77 | 1.35 |  | 3.13 | 2.4 |  | 130 | 18.7 | 0.98 | 0.74 | 1.30 |
| 50–60 | 677 | 18.5 |  | 1.64 | 1.1 |  | 128 | 18.9 | 1.04 | 0.78 | 1.38 |  | 3.11 | 2.1 |  | 125 | 18.5 | 0.94 | 0.70 | 1.25 |
| 60–70 | 473 | 12.9 |  | 1.63 | 1.2 |  | 85 | 18.0 | 0.96 | 0.69 | 1.32 |  | 3.09 | 2.2 |  | 92 | 19.5 | 0.98 | 0.72 | 1.35 |
| 70–80 | 289 | 7.9 |  | 1.68 | 1.0 |  | 51 | 17.7 | 0.94 | 0.65 | 1.38 |  | 3.20 | 2.0 |  | 61 | 21.1 | 1.04 | 0.72 | 1.50 |
| ≥80 | 189 | 5.2 |  | 1.94 | 1.2 |  | 56 | 29.6 | **1.85** | **1.25** | **2.75** |  | 3.60 | 2.2 |  | 45 | 23.8 | 1.23 | 0.81 | 1.87 |
| Total | 3668 | 100.0 |  | 1.96 | 1.4 |  | 723 | 19.7 |  |  |  |  | 3.78 | 2.7 |  | 749 | 20.4 |  |  |  |
| *p* trend |  |  |  |  |  |  |  |  | 0.17 | | |  |  |  |  |  |  | 0.78 | | |
| **Female** |  |  |  |  |  |  |  |  |  |  |  |  |  |  |  |  |  |  |  |  |
| <30 | 697 | 21.1 |  | 0.43 | 0.3 |  | 136 | 19.5 | 1.03 | 0.74 | 1.44 |  | 2.30 | 1.5 |  | 140 | 20.1 | 1.33 | 0.93 | 1.89 |
| 30–40 | 504 | 15.3 |  | 0.42 | 0.4 |  | 95 | 18.9 | 1.01 | 0.71 | 1.45 |  | 2.24 | 1.4 |  | 96 | 19.1 | 1.28 | 0.88 | 1.86 |
| 40 | 403 | 12.2 |  | 0.36 | 0.3 |  | 65 | 16.1 | 1 | Referent | |  | 3.01 | 2.3 |  | 53 | 53 | 1 | Referent | |
| 40–50 | 522 | 15.8 |  | 0.39 | 0.3 |  | 88 | 16.9 | 0.94 | 0.66 | 1.36 |  | 2.17 | 1.5 |  | 84 | 16.1 | 1.12 | 0.76 | 1.64 |
| 50–60 | 440 | 13.3 |  | 0.41 | 0.4 |  | 80 | 18.2 | 0.92 | 0.63 | 1.34 |  | 2.32 | 1.6 |  | 91 | 20.7 | 1.35 | 0.92 | 1.99 |
| 60–70 | 310 | 9.4 |  | 0.40 | 0.3 |  | 61 | 19.7 | 0.95 | 0.64 | 1.43 |  | 2.26 | 1.4 |  | 60 | 19.4 | 1.16 | 0.76 | 1.77 |
| 70–80 | 253 | 7.7 |  | 0.43 | 0.3 |  | 58 | 22.9 | 1.11 | 0.73 | 1.69 |  | 2.31 | 1.4 |  | 54 | 21.3 | 1.25 | 0.80 | 1.94 |
| ≥80 | 171 | 5.2 |  | 0.49 | 0.5 |  | 38 | 22.2 | 1.12 | 0.69 | 1.82 |  | 2.46 | 1.6 |  | 43 | 25.2 | **1.65** | **1.01** | **2.67** |
| Total | 3300 | 100.0 |  | 0.43 | 0.3 |  | 621 | 18.8 |  |  |  |  | 2.30 | 1.5 |  | 621 | 18.8 |  |  |  |
| *p* trend |  |  |  |  |  |  |  |  | 0.51 | | |  |  |  |  |  |  | 0.15 | | |
| ^§^adjusted for household income, employment condition, occupation, work shift, and weekly working hours **p* for trend was tested after excluding participants with <40 weekly working hours  CHD, Coronary Heart Disease | | | | | | | | | | | | | | | | | | | | |
|  | | | | | | | | | | | | | | | | | | | | |
